# Clinical Utility of Pre-Therapeutic [18F]FDG PET/CT Imaging for Predicting Outcomes in Breast Cancer

**DOI:** 10.3390/jcm12175487

**Published:** 2023-08-24

**Authors:** Sophia Najid, Romain-David Seban, Laurence Champion, Alexandre De Moura, Clara Sebbag, Hélène Salaün, Luc Cabel, Claire Bonneau

**Affiliations:** 1 Institut Curie, Inserm U900, 92210 Saint-Cloud, France; 2 Department of Nuclear Medicine, Institut Curie, 92210 Saint-Cloud, France; laurence.champion@curie.fr; 3 Department of Medical Oncology, Institut Curie, PSL Research University, 75005 Paris, France; alexandre.demoura@curie.fr (A.D.M.); clara.sebbag@curie.fr (C.S.); helene.salaun@curie.fr (H.S.); luc.cabel@curie.fr (L.C.); 4 UVSQ, Paris Saclay University, 92210 Saint-Cloud, France; 5 Department of Surgery, Institut Curie, 92210 Saint-Cloud, France

**Keywords:** breast cancer, neoadjuvant chemotherapy, pathological complete response, PET/CT imaging, total metabolic tumor volume

## Abstract

Background: [18F]FDG PET/CT is used for staging and could also provide information associated with clinical outcomes. The objective of this study was to determine the clinical utility of biomarkers measured using [18F]FDG PET/CT to predict the absence of pathological complete response (no-pCR) and recurrence. Methods: In this retrospective study, we included patients with non-special-type breast carcinoma who underwent [18F]FDG PET/CT before neoadjuvant chemotherapy between 2011 and 2019. Clinicopathological data were collected. Tumor SUVmax and total metabolic tumor volume (TMTV) were measured from PET images. The association between biomarkers and no-pCR was studied using logistic regression. The cut-off value was determined using the area under the ROC Curve. To predict 3-year recurrence-free survival (RFS), we used a multivariable Cox model, and the cut-off value was determined using time-dependent ROC and predictiveness curves. Results: Two hundred and eighty-six patients were included in the analysis. One hundred and twelve patients had a pCR (39.2%). The pCR rate was significantly higher in patients with a high nuclear grade (*p* < 0.01), HER2+ and TNBC subtypes (*p* < 0.01), high Ki67 (*p* < 0.01), and low TMTV (*p* < 0.01). A high TMTV value (>9.0 cm^3^) was significantly associated with no-pCR in the whole cohort (OR = 2.4, 95% CI: 1.3–4.2, *p* < 0.01). After a median follow-up of 4.5 years, 65 patients experienced recurrence and 39 patients died. High TMTV (>13.5 cm^3^) was associated with shorter RFS (HR = 4.0, 95% CI: 1.9–8.4, *p* < 0.01). Conclusion: High TMTV in pre-therapeutic imaging is associated with no-pCR and recurrence. It can help in identifying high-risk patients and be considered as an intensified or alternative adjuvant therapy for closely monitoring patients.

## 1. Introduction

Breast cancer is the most prevalent cancer in women worldwide in terms of incidence and mortality [1]. Neoadjuvant chemotherapy (NACT) was described for the first time in the 1970s with the objective of down-staging primary tumors before surgery [2]. NACT has been shown to increase the chance of breast conservation and potential survival gains [3]. More recently, the combination of neoadjuvant immunotherapy with chemotherapy in triple-negative breast cancer (TNBC) cases has shown a benefit in terms of the rate of the pathological complete response (pCR) [4] and relapse-free survival (RFS) [4]. The major prognostic factor after treatment for patients receiving NACT is the achievement of pCR [5]. Tumor size and histological grade are identified as predictive factors of pCR; however, there is no known radiological predictive factor [5,6]. The identification of more precise predictive factors is thus an area of intensive investigation that would allow the individualization of therapies and avoid some toxicities. 

Pre-therapeutic fluorine-18-fluorodeoxyglucose positron emission tomography/computed tomography ([18F]FDG PET/CT) is routinely used to rule out early metastatic disease [7,8]. The National Comprehensive Cancer Network (NCCN) guidelines currently endorse the use of [18F]FDG PET/CT for disease staging for stages IIA or IIB with N1 (1–3 axillary lymph nodes), stage IIIA with T3 or N1-N2 (1–9 axillary lymph nodes), stage IIIB, or stage IIIC. Since FDG uptake reflects tumor characteristics such as metabolic activity, [18F]FDG PET/CT could provide features associated with pCR [9,10,11] and survival [12,13,14]. Glucose metabolism has been assessed by using the maximum standard uptake value (SUVmax) as a marker of tumor aggressiveness [13]. Diao et al., in a recent meta-analysis, demonstrated its impact on recurrence-free survival (RFS) in non-metastatic breast cancer (HR = 1.96, 95% CI = 1.40–2.73) [15]. However, the value of SUVmax is only an approximate indicator of the tumor’s metabolic activity, and its measurement may be affected by the Partial Volume Effect, depending on various parameters such as size, shape, and surrounding tissue [16]. There are other imaging biomarkers on [18F]FDG PET/CT, including metabolic tumor volume (MTV) and total lesion glycolysis (TLG). Recent studies have focused on these parameters and their association with the survival and assessment of pCR [17,18,19,20,21]. These markers remain inconsistent in predicting RFS and suffer from weak reproducibility, as the thresholds identified for markers vary from one study to another depending on the chosen methodology (receiver operating characteristics (ROC) curve [18,19], predictiveness curve [17]). Similarly, both the injection protocol and the PET device used can impact the markers extracted from [18F]FDG PET/CT imaging [18,20]. Validation and standardization are essential to ensure the reproducibility and reliability of the extracted features in clinical practice.

In this study, we first investigated the ability of biomarkers by measuring pretreatment [18F]FDG PET/CT to predict the absence of pCR (no-pCR) and recurrence in comparison to standard clinicopathological variables.

## 2. Materials and Methods

### 2.1. Patients 

We conducted a retrospective, single-center study at Institut Curie Hospital, Saint-Cloud, France. Patients with histologically proven, non-special-type breast carcinoma treated with NACT (+/− HER2-targeted therapy) followed by surgery between January 2011 and December 2019 and who had a pre-therapeutic [18F]FDG PET/CT were selected. Patients were excluded due to the following criteria: (i) Patients with no measurable disease or no FDG-avid tumor, (ii) patients with another primary malignancy, (iii) patients expressing opposition to participate in medical research. This study adhered to the regulations of our Institutional Review Board (IRB DATA 230006), and we obtained a waiver of informed consent through the "no objection rule." The study was conducted in accordance with the principles outlined in the Declaration of Helsinki.

### 2.2. Clinicopathological Data

The disease’s stage was determined after a pre-therapeutic [18F]FDG PET/CT according to the 8th edition of the American Joint Committee of Cancer (AJCC) manual [22]. Characteristics of the primary tumor were obtained from the initial biopsy, including hormone receptor status (estrogen receptor (ER) and progesterone receptor (PR)), human epidermal growth factor receptor status (HER2), Ki67 proliferation index, mitotic index, histological grade, presence of vascular invasion or necrosis. Plasma levels of tumor markers (cancer antigen (CA15-3) and carcinoembryonic antigen (CEA)) were measured before treatment. Molecular subtypes were classified as follows: Luminal A (ER+/PR+/HER2−/Ki67 low), Luminal B (ER+/PR+/HER2−/Ki67 high), HER2 protein overexpression (HER2+/ER−/PR−), and Basal-like (Triple Negative Breast Cancer (TNBC): ER−/PR−/HER2−) [23]. We considered Ki67 to be high when values exceeded 20%. Hormone receptor status was considered positive for values above 10% [24].

### 2.3. Treatment, Pathological Complete Response, and Surveillance

Treatment decisions were made after a multidisciplinary meeting and in accordance with European guidelines [24]. All patients were treated with NACT +/− HER2-targeted therapy followed by breast conservation or total surgery and axillary exploration (axillary dissection or sentinel lymph node) when indicated. The pathological complete response (pCR) was defined on the surgical tissue as the absence of residual tumor in the breast and lymph nodes ypT0/ypN0 (Residual Cancer Burden = 0) [25,26]. The pCR rate (ypT0/ypN0) was defined as the percentage of participants without residual invasive and in situ cancer upon evaluation of the complete resected breast specimen and all sampled regional lymph nodes. Patients received adjuvant therapy (chemotherapy, hormonal therapy, HER2-targeted therapy, radiotherapy) if it was recommended. After treatment, patients were followed regularly (clinical examination, annual mammography, tumor markers) according to international recommendations. In cases of suspected recurrence, a biopsy of the lesion was recommended, and a thorough imaging assessment was requested, including a CT scan or MRI. An [18F]FDG PET/CT scan was only performed for restaging (when recurrence was confirmed).

### 2.4. PET/CT Imaging

[18F]FDG PET/CT scans were performed according to EANM guidelines [27]. Patients fasted for at least 6 hours before the examination to ensure that their plasma glucose level was below 10 mmol/L. Scanning was performed from the skull base to the proximal femur using two different devices: an analog PET (n = 201), General Electric Discovery-690, and a digital PET (n = 85), Philips Vereos. PET images were reconstructed using the following iterative algorithms: Vue Point FX algorithm, time of flight –TOF reconstruction, matrix 256 × 256, 2 iterations, 24 subsets, post-filter 6.4mm (GE Discovery-690) and OSEM algorithm, time of flight –TOF reconstruction, matrix 288 × 288, 3 iterations, 5 subsets, post-filter 2mm (Philips Vereos). Given the heterogeneity induced by the two PET devices, we decided to perform an analysis with two distinct subgroups (cohort 1 = analog PET versus cohort 2 = digital PET). PET images were converted to standard uptake value (SUV) by normalizing with the patient’s body weight. Two nuclear medicine physicians performed the analysis of the images without any information about the patient’s history, pathology, imaging characteristics, or clinical outcomes. Lesions were segmented using the PET tumor segmentation tool in Philips IntelliSpace Portal 9.0. Primary tumor and regional lymph nodes were delineated on images, following EANM guidelines [27]. SUVmax was defined as the highest SUV among all detected lesions, including the primary tumor and any metastatic lymph nodes. Total metabolic tumor volume (TMTV) was calculated as the sum of the metabolic tumor volume of all lesions.

### 2.5. Outcomes Measures

Endpoints were the association between imaging biomarkers and no-pCR (defined above) and survival using recurrence-free survival (RFS), determined by measuring the duration from the initial treatment received to the occurrence of disease relapse or death from any cause. In addition, the censoring date was set as the last recorded instance when the patient was known to be alive. We considered RFS at 3 years, consistent with the median follow-up of the cohort. Follow-up time was calculated as the time from pre-therapeutic [18F]FDG PET/CT to the last visit.

### 2.6. Statistical Analysis

Means, minima, and maxima were used for descriptive analysis. Differences between the imaging biomarkers and clinico-pathological markers between cohort 1 (analog PET) and cohort 2 (digital PET) were evaluated using Fisher’s exact test in cases of qualitative variables and Student’s test in cases of quantitative variables. The distribution of imaging biomarkers between patients who achieved pCR and those who did not (no-pCR) was compared using the Mann–Whitney–Wilcoxon test. 

If a significant relationship existed between imaging biomarkers (SUVmax and TMTV) and pCR, they were then dichotomized for further analysis. The cut-off value was determined by the area under the ROC Curve. Factors associated with no-pCR were tested using a logistic regression model. The final multivariate model was obtained after a step-by-step selection procedure. The model was initially run on the whole cohort; then, the same process was carried out on cohort 1 and cohort 2.

The prognostic impact of all factors measured at diagnosis was studied using Cox proportional hazards models for survival in the whole cohort. We used two different methods to determine the relevant cut-off value (considering a 3-year time horizon): (i) predictiveness curves and (ii) time-dependent ROC curves. Found thresholds were used to binarize continuous variables to produce Kaplan–Meier survival curves. The *p* values obtained are those of the log rank test. The final prognostic model for 3-year survival was constructed using a step-by-step selection procedure. The likelihood ratio test (LRT) for the added prognostic value of imaging biomarkers was obtained by comparing the log-likelihoods of the multivariable prognostic models with and without those markers (chi-square test).

Finally, given the evolving therapeutic landscape, we performed a subgroup analysis on the TNBC subtype using the thresholds obtained in the whole cohort. 

The alpha risk was fixed at 5%, and a difference was considered significant if the *p*-value was lower than 0.05. Statistical analysis was carried out using R software (version 4.2.2, R Core Team (2022), R Foundation for Statistical Computing, Vienna, Austria)

## 3. Results

### 3.1. Patients’ Characteristics 

**Figure 1:**
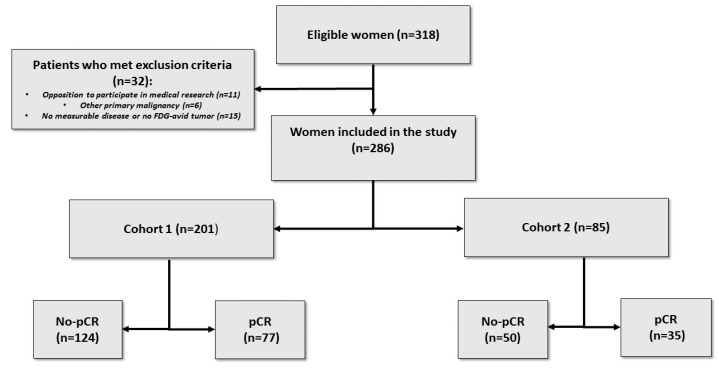
Flow Chart.

Between January 2011 and December 2019, 320 patients had their breast cancer managed using NACT followed by surgery, as well as receiving an [18F]FDG PET/CT before treatment. Thirty-two patients were excluded for failing to meet the inclusion criteria: 11 patients expressed opposition to participating in the medical research, 6 had another primary malignancy, and 15 had no measurable disease on imaging (Figure 1). In the whole cohort (N = 286), 201 patients were staged with an analog [18F]FDG PET/CT, these patients constituted cohort 1 and 85 with a digital [18F]FDG PET/CT (cohort 2).

Overall, pathological complete response (pCR) after NACT was obtained in 112 breast cancers (39.2%). The mean age of the patients was 49 years (±12.4). More than one-half of patients had histologically proven regional lymph node metastasis (58%). On histopathological assessment, 9.8% (n = 28) of tumors were classified as Luminal A, 24.8% (n = 71), Luminal B HER2-, 23.8% (68) HER2+ (Luminal B HER2 + (n = 39), HER2 enriched (n = 29)), and 41.6% (n = 119) Basal-like (TNBC) (Supplemental Figure S1). All patients with HER2+ breast cancer received HER2-targeted therapy in combination with NACT and 53.5% received endocrine therapy. Most patients (96.5%) received external radiotherapy after surgery and only 23.9% received adjuvant chemotherapy. Clinicopathological characteristics were similarly distributed in both cohorts. However, the mean maximum standardized uptake value (SUVmax) was higher in cohort 2, and there were significantly more patients who received chemotherapy and HER2-targeted therapy as adjuvant therapy (Table 1). The TNBC subtype appeared to have a higher SUVmax than the luminal and HER2+ subtypes. However, TMTV appeared to be similar for the subtypes studied (Supplemental Table S1).

### 3.2. Association with Pathological Complete Response

#### 3.2.1. Relationship between Biomarkers and pCR 

The pCR rate was significantly higher in patients with a high nuclear grade (*p* < 0.01), HER2+ and TNBC subtypes (*p* < 0.01), high Ki67 (*p* < 0.01), and low TMTV (*p* < 0.01). No relationship with SUVmax was demonstrated (Supplemental Table S2). 

#### 3.2.2. Determination of Cut-Off Value of TMTV to Predict pCR 

The cut-off value of TMTV for pCR was determined using the receiver operating ROC curve calculated using the Youden index for the areas under the curve (AUC) (Supplemental Figure S2). The best cut-off value was 9.0 cm^3^ (AUC = 0.64, Sensitivity (Se) = 0.59, Specificity (Sp) = 0.66).

#### 3.2.3. Univariate and Multivariate Analyses of pCR Including TMTV (High versus Low)

In the univariate analysis (Table 2), grades 1–2, T stages 3–4, Ki67 < 20%, Luminal subtype, and lymph node involvement, as well as TMTV higher than 9.0 cm^3^, were significantly associated with no-pCR, whereas this relationship was not observed for age (<40 years vs. ≥40 years) and vascular invasion. In the multivariate regression analysis, high TMTV (OR = 2.4 95% CI: 1.3–4.2 *p* value < 0.01) remained an independent and statistically significant predictive factor for no-pCR.

By performing the same analysis on cohort 1 (analog PET) and cohort 2 (digital PET), we observed similar results with different cut-off values depending on the machine used (Supplemental Figures S3 and S4). The optimal cut-off value for cohort 1 was equal to 6.3 cm^3^ (AUC = 0.61, Se = 0.45, Sp = 0.78), while, for cohort 2, it was 2.7 cm^3^ (AUC = 0.63, Se = 0.39, Sp = 0.86). A high TMTV remained an independent and statistically significant prognostic factor for no-pCR in both cohorts (cohort 1: OR = 2.5, 95% CI: 1.2–5.3; cohort 2: OR = 3.6, 95% CI: 1.1–12.3) (Supplemental Tables S3 and S4).

### 3.3. Association with Recurrence-Free Survival

#### 3.3.1. Determination of the Best Cut-Off Value of TMTV to Predict 3-Year RFS

The best TMTV cut-off value predicting 3-year RFS according to the time-dependent ROC curve was 13.5 cm^3^ (Supplemental Figure S5). When applying this value, 118 (41%) patients had a high TMTV.

According to the predictiveness curve, the best cut-off value was 28.1 cm^3^ (Supplemental Figure S6). When applying this value, only 65 (23%) had a high TMTV.

#### 3.3.2. Survival Analysis 

**Figure 2:**
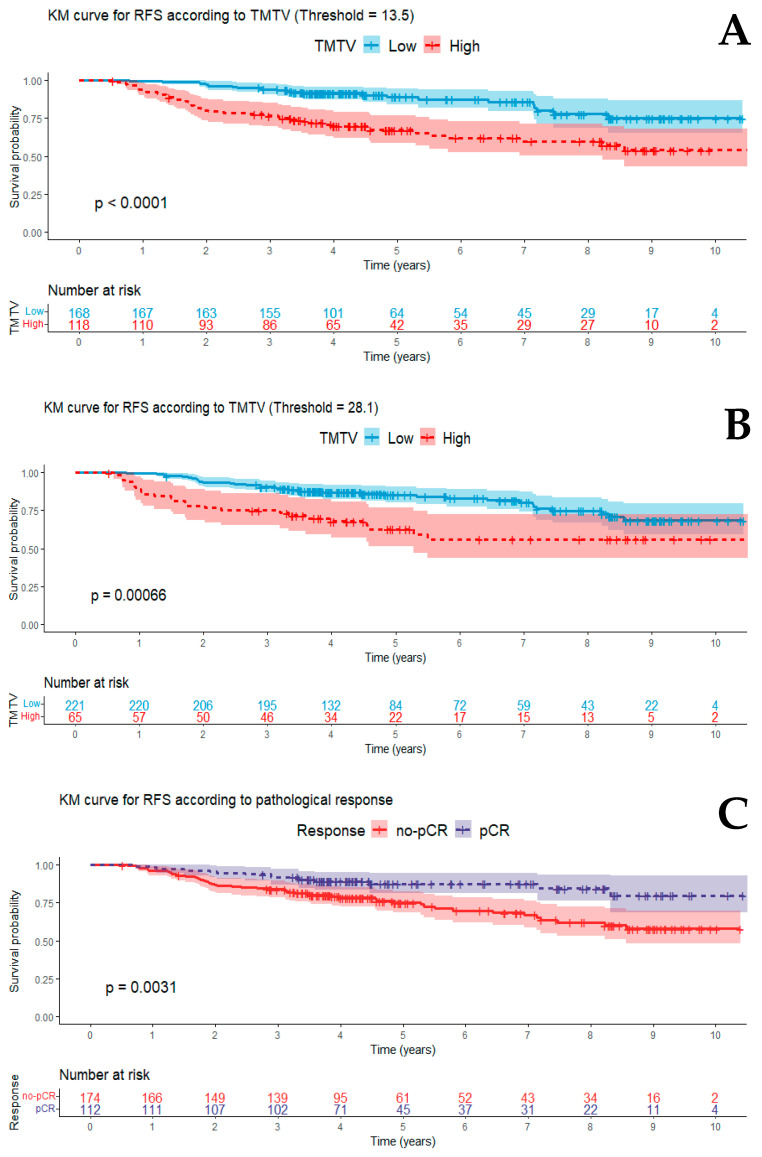
Kaplan–Meier curves according to TMTV with a cut-off value of 13.5 cm^3^ (**A**), of 28.1 cm^3^ (**B**), and according to pathological complete response (**C**). The *p* values obtained are those of the log rank test.

During a median follow-up of 4.5 years (95% CI: 3.7–7.8), 65 patients experienced recurrence (Luminal: 24.2%, HER2+: 1.2%, TNBC: 25.2%) and 39 patients died (Luminal: 15.1%, HER2+: 5.9%, TNBC: 16.8%). The median RFS was 4.3 years (95% CI: 3.5–7.1). In the univariate analysis, lymph node involvement (N+), no-pCR, and high TMTV were significantly associated with a higher 3-year risk of recurrence. RFS decreased when TMTV was high regardless of the threshold used (Figure 2). In multivariate analysis, only high TMTV (HR = 4.0 95% CI: 1.9–8.4) remained an independent and statistically significant prognostic factor for 3-year RFS (Table 3). These results were also significant when applying the threshold obtained using the predictiveness curve (HR = 2.6 95% CI: 1.3–5.0) (Supplemental Table S5). Finally, TMTV added significant prognostic values to the multivariable model obtained when the cut-off value was 13.5 cm^3^ but not when the cut-off was 28.1 cm^3^ (*p* < 0.01 and *p* = 0.1 respectively, Supplemental Table S6).

### 3.4. Subgroup Analysis: Triple Negative Breast Cancer 

Overall, 119 patients in the cohort had a TNBC molecular subtype (41.6%). Among the 119 patients, the rate of pCR was 48% (57/119).

#### 3.4.1. Association with Pathological Complete Response 

To identify the ability of TMTV to predict pCR for TNBC breast cancer, we used thresholds identified in the whole cohort (e.g., 3.2.2. TMTV > 9.0 cm^3^ vs. ≤ 9.0 cm^3^). In the univariate analysis, tumor size and TMTV were significantly associated with no-pCR. In multivariate analysis, only a high TMTV remained an independent prognostic factor (OR = 3.6 95% CI: 1.5–8.6) (Table 4). 

#### 3.4.2. Association with Recurrence-Free Survival

The median follow-up was 4.3 years (95% CI: 3.5–6.8). During this period, 20 (16,8%) patients with TNBC breast cancer died, and 30 patients (25,2%) experienced recurrence. The median RFS was 4.2 years (95% CI: 3.5–6.7). To study the ability of TMTV to predict 3-year, recurrence-free survival, we used thresholds identified using the time-dependent ROC curve conducted on the whole cohort (e.g., 3.3.1 TMTV > 13.5 cm^3^ vs. ≤ 13.5 cm^3^). In the multivariate Cox model, a high TMTV was significantly associated with RFS (HR = 3.1 95% CI: 1.2–7.9) (Table 5, Supplemental Figure S7). 

## 4. Discussion

**Figure 3:**
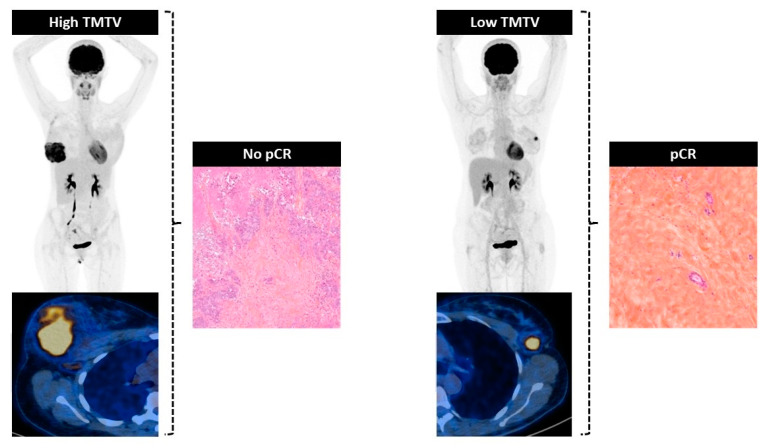
Association between the value of TMTV on pre-therapeutic [18F]FDG PET/CT and pathological response on the surgical specimen after surgery.

In the present study, we demonstrated the clinical utility of pre-therapeutic [18F]FDG PET/CT imaging for predicting clinical outcomes after NACT in breast cancer patients. We have shown that a high total metabolic tumor volume (TMTV) before NACT is a critical biomarker associated with no-pCR (Figure 3) and recurrence (3-RFS) in this retrospective cohort of 286 patients. Given the evolving therapeutic landscape with the pivotal Keynote-552 trial [4], we focused on TNBC patients and confirmed its clinical utility for the early identification of patients with a high risk of no-pCR and recurrence at 3 years.

The total metabolic tumor volume reflects the tumor burden along with a metabolically active lesion within the breast +/− regional lymph node(s). Our study demonstrated a strong association between TMTV and no-pCR. Few studies have previously examined this relationship. Le Marignier et al. [12], in their cohort of 171 ER+ HER2- breast cancers, found that none of the biomarkers used were predictive of pCR. In contrast, Higushi et al. [21] showed in their study that a low metabolic tumor volume (MTV) in pre-therapeutic imaging within the tumor was associated with pCR (OR = 0.30, 95% CI: 0.11–0.84, *p* = 0.02). However, the authors only focused on MTV within the tumor and not TMTV. Similarly, Urso et al. [18] showed that non-responding patients with Luminal B tumors had a higher median MTV within the tumor compared to responders (7.3 ± 4.2 cm^3^ versus 3.5 ± 2.5 cm^3^). The optimal TMTV cut-off value for predicting pCR highlighted by the authors was higher than that in our study (17.7 cm^3^, AUC = 0.73 versus 9.0 cm^3^, AUC = 0.64).

Our results confirm the prognostic value of TMTV on pre-therapeutic [18F]FDG PET/CT. Our team previously showed a significant association between a high pre-therapeutic TMTV and recurrence at 5 years in a cohort of 303 patients with treated early-stage breast cancer, including 51% of patients who received NACT [17]. Similar results have been demonstrated in a cohort of 40 patients treated with NACT but with a different TMTV cut-off than that calculated in our study (19.3 cm^3^ versus 13.5 cm^3^) [19]. Regardless of the threshold used, a high TMTV is a prognostic marker for recurrence, and it is nevertheless interesting to consider this marker to decide on therapeutic management after NACT, especially when pCR is not achieved, while waiting for a validated threshold on a larger scale.

Regarding TNBC, various studies have shown that they are more sensitive to NACT than luminal-type tumors [3,28]. Identifying biomarkers that can reliably select high- and low-risk subsets of patients at the time of surgery is crucial for making treatment decisions. Through our subgroup analysis, we were able to highlight the prognostic value of the TMTV biomarker for TNBC. This is in line with the findings of Urso et al. [18]. In their study, TMTV in responders who died during follow-up was significantly higher than in living patients (12.8 cm^3^ ± 15.2 cm^3^ versus 43.2 cm^3^ ± 26.7 cm^3^, *p* = 0.01). However, in their study, no threshold value was found to predict pCR, maybe due to the small number of TNBC patients in their group (32 versus 119 in our cohort). Recently, it has been recommended to combine immunotherapy (anti-PD1) with NACT in TNBC cases [4]. In the future, it would be interesting to explore if TMTV on pre-therapeutic [18F]-FDG PET/CT could predict pCR in the specific population of TNBC patients undergoing NACT associated with immunotherapy. It would be interesting to define risk groups of patients in order to identify those who would benefit from combined treatment.

PET imaging could provide prognostic biomarkers in oncology but their specific impact is assessed using diverse methodologies. First, the method of determining optimal threshold values for survival analysis is very heterogeneous and, subsequently, leads to a lack of reproducibility/external validity. For instance, there are two basic statistical approaches commonly used to determine optimal threshold values for survival analysis. The first is to evaluate the biomarker effect on risk and disease outcomes (logistic regression, Cox models, Kaplan–Meier analysis). The second is to determine the biomarker performance using classification measures (sensitivity, specificity, predictive values, and time-dependent AUC within ROC curves). Results may vary depending on the approach chosen, and there is currently no consensus regarding which should be preferred. This is why we chose to test the prognostic value of TMTV with the two approaches. On one hand, we used ROC curves, which are used to study the discriminative and predictive power of TMTV without taking into account risks [29]. On the other hand, we used predictiveness curves, which provide information about risks and classification performance [30]. In our study, TMTV was significantly associated with 3-year RFS regardless of the method used for determining thresholds (time-dependent AUC/ROC and predictiveness curves). Second, previous papers have suggested a prognostic impact of PET biomarkers; however, the acquisition of PET images may have certainly impacted the reproducibility/external validity of such results. We have thus performed our analysis using two distinct cohorts based on the PET device (analog/cohort 1 vs. digital/cohort 2). Again, TMTV was significantly associated with clinical outcomes regardless of the nature of the PET device. These findings strengthen the predictive and prognostic value of TMTV on pre-therapeutic [18F]FDG PET/CT for early-stage breast cancer.

The main strength of our study is the sample size. Some limitations should be emphasized, particularly those inherent to the retrospective and monocentric nature of the study. From a statistical point of view, we have tested a limited number of variables compared to the number of events in our data to avoid overfitting (pCR analysis with logistic regression: 14 events/variable and recurrence analysis with Cox regression: 7 events/variable). Although the final results are conclusive, a prospective study with a larger sample size should be conducted to better demonstrate these results. Finally, we have included patients over a period of 7 years; it is plausible that advancements in surgical, radiation, and medical oncology may lead to better patient outcomes.

## 5. Conclusions

The evaluation of metabolic tumor burden using TMTV on [18F]FDG PET/CT before NACT could help identify high-risk patients who are more likely to experience no-pCR and/or recurrence. While larger prospective studies are warranted to validate these findings, this imaging feature should be taken into account to guide clinical decision-making, especially in the specific population with TNBC undergoing neoadjuvant therapy. 

## Figures and Tables

**Table 1:** Description of baseline characteristics and comparison between the two cohorts.

All Patients N = 286	Overall N = 286	Cohort 1 N = 201	Cohort 2 N = 85	
Mean (±SD), n (%)				*p* Value
Clinicopathological characteristics				
Age (years)	49.1 (±12.4)	48.7 (±12.65)	50.0 (±11.8)	0.39
pCR	112 (39.2)	77 (38.3)	35 (41.2)	0.65
**TNM**				
T stage				0.14
1	26 (9.1)	15 (7.5)	11 (12.9)	
2	162 (56.6)	113 (56.2)	49 (57.6)	
3	75 (26.2)	59 (29.4)	16 (18.8)	
4	23 (8.0)	14 (7.0)	9 (10.6)	
N+	166 (58.0)	124 (61.7)	42 (49.4)	0.07
**Subtype**				0.43
Luminal A	28 (9.8)	43 (21.4)	25 (29.4)	
Luminal B HER2-	71 (24.8)	20 (10.0)	8 (9.4)	
HER2+ *	68 (23.8)	54 (26.9)	17 (20.0)	
TNBC	119 (41.6)	84 (41.8)	35 (41.2)	
**Histologic parameters**				
Ki67	49.8 (±24.2)	49.7 (±24.1)	50.1 (±24.6)	0.21
Vascular invasion	25 (8.7)	20 (10.0)	8 (9.4)	0.27
**Grade**				0.79
I/II	95 (33.2)	68 (33.8)	27 (31.8)	
III	191 (66.8)	133 (66.2)	58 (68.2)	
**Mitotic index**				0.64
1	62 (22.2)	44 (22.7)	18 (21.2)	
2	97 (34.8)	64 (33.0)	33 (38.8)	
3	120 (43.0)	86 (44.3)	34 (40.0)	
**Tumor markers (ng/mL)**				
CEA	2.76 (±6.0)	2.89 (±6.6)	2.1 (±1.6)	0.53
CA 15-3	24.4 (±43.2)	26.2 (±42.8)	22.8 (±22.5)	0.35
**PET imaging characteristics**				
**SUVmax**	**11.7 (±6.4)**	**11.1 (±6.2)**	**13.14 (±6.7)**	**0.01**
TMTV	24.4 (±43.2)	27.4 (±42.8)	17.3 (±43.6)	0.07
**Treatment**				
**Neoadjuvant**				
HER2-targeted therapy	68 (23.8)	43 (21.4)	25 (29.4)	0.19
**Adjuvant**				
Radiotherapy	276 (96.5)	193 (96)	83 (97.6)	0.74
**Chemotherapy**	**68 (23.8)**	**30 (14.19)**	**38 (45.2)**	**<0.01**
**HER2-targeted therapy**	**32 (11.2)**	**16 (7.69)**	**16 (18.82)**	**0.01**
Endocrine therapy	153 (53.5)	104 (51.7)	49 (57.6)	0.43

Abbreviations: tumor (T), node involvement (N), triple negative breast cancer (TNBC), cancer antigen 15-3 (CA15-3), carcinoembryonic antigen (CEA), maximum standardized uptake value (SUVmax), total metabolic tumor volume (TMTV), HER2+ *: Enriched and Luminal B HER2+.

**Table 2:** Logistic regression analysis of biomarkers associated with no pathological complete response in the whole cohort.

Factor Associated with No-pCR after NACT				
N = 286	Univariate		Multivariate	
Events = 112	OR (95% CI)	*p* Value	OR (95% CI)	*p* Value
Age < 40 years (vs. ≥40)	0.6 (0.4–1.1)	0.09	0.7 (0.4–1.2)	0.16
T stage 3–4 (vs. 1–2)	2.1 (1.2–3.5)	<0.01	1.6 (0.9–3.1)	0.13
N+ (vs. N−)	2.19 (1.3–3.6)	<0.01	-	-
**Subtype**			**-**	**<0.01**
Luminal	1.0 (Reference)	-	-	-
HER2+	0.3 (0.1–0.5)	<0.01	0.3 (0.1–0.6)	-
TNBC	0.3 (0.2–-0.5)	<0.01	0.4 (0.2–0.7)	-
Vascular invasion (yes vs. no)	1.7 (0.7–4.6)	0.20	-	-
Histologic grade 3 (vs. 1–2)	0.4 (0.2–0.7)	<0.01	-	-
**Ki67 ≥ 20% (vs. <20%)**	**0.3 (0.1–0.6)**	**<0.01**	**0.3 (0.1** **–** **0.7)**	**<0.01**
**TMTV > 9.0 cm^3^ (vs. ≤9.0 cm^3^)**	**2.9 (1.8–4.9)**	**<0.01**	**2.4 (1.3–4.2)**	**<0.01**

Abbreviations: odd ratio (OR), confidence interval (CI), pathological complete response (pCR), neoadjuvant chemotherapy (NACT), tumor (T), node involvement (N), triple negative breast cancer (TNBC), total metabolic tumor volume (TMTV).

**Table 3:** Prognostic significance of biomarkers for 3-year RFS in univariate and multivariate analyses (Cox models) with threshold values of TMTV equal to 13.5 cm^3^.

3-Year Recurrence-Free Survival				
n = 286	Univariate		Multivariate	
Events = 65	HR (95% CI)	*p* Value	HR (95% CI)	*p* Value
Age < 40 years (vs. ≥40)	1.1 (0.5–2.3)	0.78	-	-
pCR	0.5 (0.2–0.9)	0.04	0.6 (0.3–1.2)	0.14
T stage 3–4 (vs. 1–2)	1.51(0.8–2.9)	0.21	-	-
N+ (vs. N−)	2.4 (1.1–5.1)	0.02	**-**	**-**
Molecular subtype				
Luminal	1.0 (Reference)	-	-	-
HER2+	0.3 (0.1–1.2)	0.09	-	-
TNBC	1.6 (0.8–3.2)	0.20	-	-
Vascular invasion (yes vs. no)	1.7 (0.6–4.4)	0.26	**-**	**-**
Histologic grade 3 (vs. 1–2)	0.8 (0.4–1.6)	0.55	-	-
Ki67 ≥ 20% (vs. <20%)	2.4 (0.7–7.7)	0.15	2.8 (0.8–9.0)	0.09
**TMTV > 13.5 cm^3^ (vs. ≤13.5 cm^3^)**	**4.4 (2.1–9.1)**	**<0.01**	**4.0 (1.9** **–8.4)**	**<0.01**

Abbreviations: hazard ratio (HR), confidence interval (CI), pathological complete response (pCR), tumor (T), node involvement (N), triple negative breast cancer (TNBC), total metabolic tumor volume (TMTV).

**Table 4:** Logistic regression analysis of biomarkers associated with no-pCR for TNBC.

Factor Associated with no-pCR after NACT				
n = 119	Univariate		Multivariate	
Events = 57	OR (95% CI)	*p* Value	OR (95% CI)	*p* Value
Age < 40 years (vs. ≥40)	1.0 (0.5–2.3)	0.99	-	-
T stage 3–4 (vs. 1–2)	4.1 (1.8–10.3)	<0.01	2.2 (0.8–6.0)	0.12
N+ (vs. N−)	1.9 (0.9–4.1)	0.07	-	-
Vascular invasion (yes vs. no)	1.7 (0.5–6.7)	0.40	-	-
Histologic grade 3 (vs. 1–2)	0.5 (0.2–1.5)	0.30	-	-
Ki67 ≥ 25% (vs. <25%)	0.3 (0.02–2.8)	0.40	**-**	**-**
**TMTV > 9.0 cm^3^ (vs. ≤9.0 cm^3^)**	**4.9 (2.3–11.0)**	**<0.01**	**3.6 (1.5–8.6)**	**<0.01**

Abbreviations: odd ratio (OR), confidence interval (CI), pathological complete response (pCR), neoadjuvant chemotherapy (NACT), tumor (T), node involvement (N)), total metabolic tumor volume (TMTV).

**Table 5:** Prognostic significance of biomarkers for 3-year RFS in univariate and multivariate analyses (Cox models) with a threshold value of TMTV equal to 13.5 cm^3^ for TNBC.

3-Year Recurrence-Free Survival				
n = 119	Univariate		Multivariate	
Events = 30	HR (95% CI)	*p* Value	HR (95% CI)	*p* Value
Age < 40 years (vs. ≥40)	1.5 (0.5–4.1)	0.42	-	-
pCR	0.3 (0.1–0.8)	0.01	0.4 (0.1–1.1)	0.07
T stage 3–4 (vs. 1–2)	1.4 (0.6–3.4)	0.41	-	-
N+ (vs. N−)	2.9 (1.2–7.3)	0.02	**-**	**-**
Vascular invasion (yes vs. no)	1.8 (0.5–6.0)	0.36	**-**	**-**
Histologic grade 3 (vs. 1–2)	0.5 (0.2–1.5)	0.22	-	-
Ki67 ≥ 25% (vs. <25%)	0.7 (0.1–5.5)	0.77	-	-
**TMTV > 13.5 cm^3^ (vs. ≤13.5 cm^3^)**	**4.0 (1.6–9.8)**	**< 0.01**	**3.1 (1.2** **–7.9)**	**0.01**

Abbreviations: hazard ratio (HR), confidence interval (CI), pathological complete response (pCR), tumor (T), node involvement (N)), total metabolic tumor volume (TMTV).

## Data Availability

Datasets are available upon request.

## Supplementary Materials

The following supporting information can be downloaded at: https://www.mdpi.com/article/10.3390/jcm12175487/s1, Supplemental Figure S1: Distribution of the different molecular subtypes. Supplemental Table S1: Distribution of PET imaging biomarkers. Supplemental Table S2: Comparison of clinicopathological characteristics and PET imaging characteristics between patients with a pathological complete response and no pathological complete response. Supplemental Figure S2: ROC curve to determine the best cut-off value of TMTV to predict pCR in the whole cohort. Supplemental Figure S3: ROC curve to determine the best cut-off value of TMTV to predict pCR in cohort 1. Supplemental Table S3: Logistic regression analysis of biomarkers associated with no-pCR in cohort 1. Supplemental Figure S4: ROC curve to determine the best cut-off value for TMTV to predict pCR in cohort 2. Supplemental Table S4: Logistic regression analysis of biomarkers associated with no-pCR in cohort 2. Supplemental Figure S5: Time-dependent ROC curve for TMTV to predict 3-year RFS. Supplemental Figure S6: Predictiveness curve for TMTV for 3-year RFS. Supplemental Table S5: Prognostic significance of biomarkers for 3-year RFS in univariate and multivariate analyses (Cox models) with threshold values of TMTV equal to 28.1 cm^3^. Supplemental Table S6: Test for additional prognostic value of TMTV. Supplemental Figure S7: Kaplan–Meier curves according to molecular subtype.

## References

[1] Sung H., Ferlay J., Siegel R.L., Laversanne M., Soerjomataram I., Jemal A., Bray F. (2021). Global Cancer Statistics 2020: GLOBOCAN Estimates of Incidence and Mortality Worldwide for 36 Cancers in 185 Countries. CA Cancer J. Clin..

[2] Rubens R.D., Sexton S., Tong D., Winter P.J., Knight R.K., Hayward J.L. (1980). Combined chemotherapy and radiotherapy for locally advanced breast cancer. Eur. J. Cancer.

[3] Early Breast Cancer Trialists’ Collaborative Group (EBCTCG) (2018). Long-term outcomes for neoadjuvant versus adjuvant chemotherapy in early breast cancer: Meta-analysis of individual patient data from ten randomised trials. Lancet Oncol..

[4] Schmid P., Cortes J., Dent R., Pusztai L., McArthur H., Kümmel S., Bergh J., Denkert C., Park Y.H., Hui R. (2022). Event-free survival with pembrolizumab in early triple-negative breast cancer. N. Engl. J. Med..

[5] Cortazar P., Zhang L., Untch M., Mehta K., Costantino J.P., Wolmark N., Bonnefoi H., Cameron D., Gianni L., Valagussa P. (2014). Pathological complete response and long-term clinical benefit in breast cancer: The CTNeoBC pooled analysis. Lancet.

[6] Parker J.S., Mullins M., Cheang M.C., Leung S., Voduc D., Vickery T., Davies S., Fauron C., He X., Hu Z. (2009). Supervised risk predictor of breast cancer based on intrinsic subtypes. J. Clin. Oncol..

[7] Ko H., Baghdadi Y., Love C., Sparano J.A. (2020). Clinical Utility of 18F-FDG PET/CT in Staging Localized Breast Cancer Before Initiating Preoperative Systemic Therapy. J. Natl. Compr. Cancer Netw..

[8] Hyland C.J., Varghese F., Yau C., Beckwith H., Khoury K., Varnado W., Hirst G.L., Flavell R.R., Chien A.J., Yee D. (2020). Use of 18F-FDG PET/CT as an Initial Staging Procedure for Stage II-III Breast Cancer: A Multicenter Value Analysis. J. Natl. Compr. Cancer Netw..

[9] Groheux D., Mankoff D., Espié M., Hindié E. (2016). ¹⁸F-FDG PET/CT in the early prediction of pathological response in aggressive subtypes of breast cancer: Review of the literature and recommendations for use in clinical trials. Eur. J. Nucl. Med. Mol. Imaging.

[10] Groheux D. (2014). Predicting pathological complete response in breast cancer early. Lancet Oncol..

[11] Li P., Wang X., Xu C., Liu C., Zheng C., Fulham M.J., Feng D., Wang L., Song S., Huang G. (2020). ^18^F-FDG PET/CT radiomic predictors of pathologic complete response (pCR) to neoadjuvant chemotherapy in breast cancer patients. Eur. J. Nucl. Med. Mol. Imaging.

[12] Lemarignier C., Martineau A., Teixeira L., Vercellino L., Espié M., Merlet P., Groheux D. (2017). Correlation between tumour characteristics, SUV measurements, metabolic tumour volume, TLG and textural features assessed with ^18^F-FDG PET in a large cohort of oestrogen receptor-positive breast cancer patients. Eur. J. Nucl. Med. Mol. Imaging.

[13] Groheux D., Giacchetti S., Moretti J.-L., Porcher R., Espié M., Lehmann-Che J., de Roquancourt A., Hamy A.-S., Cuvier C., Vercellino L. (2011). Correlation of high ^18^F-FDG uptake to clinical, pathological and biological prognostic factors in breast cancer. Eur. J. Nucl. Med. Mol. Imaging.

[14] Nishimukai A., Inoue N., Kira A., Takeda M., Morimoto K., Araki K., Kitajima K., Watanabe T., Hirota S., Katagiri T. (2017). Tumor size and proliferative marker geminin rather than Ki67 expression levels significantly associated with maximum uptake of 18F-deoxyglucose levels on positron emission tomography for breast cancers. PLoS ONE.

[15] Diao W., Tian F., Jia Z. (2018). The prognostic value of SUVmax measuring on primary lesion and ALN by 18F-FDG PET or PET/CT in patients with breast cancer. Eur. J. Radiol..

[16] Soret M., Bacharach S.L., Buvat I. (2007). Partial-volume effect in PET tumor imaging. J. Nucl. Med..

[17] Seban R.D., Rouzier R., Latouche A., Deleval N., Guinebretiere J.M., Buvat I., Bidard F.C., Champion L. (2021). Total metabolic tumor volume and spleen metabolism on baseline [18F]-FDG PET/CT as independent prognostic biomarkers of recurrence in resected breast cancer. Eur. J. Nucl. Med. Mol. Imaging.

[18] Urso L., Evangelista L., Alongi P., Quartuccio N., Cittanti C., Rambaldi I., Ortolan N., Borgia F., Nieri A., Uccelli L. (2022). The Value of Semiquantitative Parameters Derived from 18F-FDG PET/CT for Predicting Response to Neoadjuvant Chemotherapy in a Cohort of Patients with Different Molecular Subtypes of Breast Cancer. Cancers.

[19] Jiménez-Ballvé A., García García-Esquinas M., Salsidua-Arroyo O., Serrano-Palacio A., García-Sáenz J.A., Ortega Candil A., Fuentes Ferrer M.E., Rodríguez Rey C., Román-Santamaría J.M., Moreno F. (2016). Prognostic value of metabolic tumour volume and total lesion glycolysis in 18F-FDG PET/CT scans in locally advanced breast cancer staging. Rev. Esp. Med. Nucl. Imagen Mol..

[20] Farrugia M.K., Wen S., Jacobson G.M., Salkeni M.A. (2018). Prognostic factors in breast cancer patients evaluated by positron-emission tomography/computed tomography before neoadjuvant chemotherapy. World J. Nucl. Med..

[21] Higuchi T., Fujimoto Y., Ozawa H., Bun A., Fukui R., Miyagawa Y., Imamura M., Kitajima K., Yamakado K., Miyoshi Y. (2019). Significance of metabolic tumor volume at baseline and reduction of mean standardized uptake value in 18F-FDG-PET/CT imaging for predicting pathological complete response in breast cancers treated with preoperative chemotherapy. Ann. Surg. Oncol..

[22] Cserni G., Chmielik E., Cserni B., Tot T. (2018). The new TNM-based staging of breast cancer. Virchows Arch..

[23] Goldhirsch A., Wood W.C., Coates A.S., Gelber R.D., Thürlimann B., Senn H.J., Panel members (2011). Strategies for subtypes–dealing with the diversity of breast cancer: Highlights of the St. Gallen international expert consensus on the primary therapy of early breast cancer 2011. Ann. Oncol..

[24] Cardoso F., Kyriakides S., Ohno S., Penault-Llorca F., Poortmans P., Rubio I.T., Zackrisson S., Senkus E., ESMO Guidelines Committee (2019). Early breast cancer: ESMO Clinical Practice Guidelines for diagnosis, treatment and follow-up. Ann. Oncol..

[25] Provenzano E., Bossuyt V., Viale G., Cameron D., Badve S., Denkert C., MacGrogan G., Penault-Llorca F., Boughey J., Curigliano G. (2015). Standardization of pathologic evaluation and reporting of postneoadjuvant specimens in clinical trials of breast cancer: Recommendations from an international working group. Mod. Pathol..

[26] Symmans W.F., Peintinger F., Hatzis C., Rajan R., Kuerer H., Valero V., Assad L., Poniecka A., Hennessy B., Green M. (2007). Measurement of residual breast cancer burden to predict survival after neoadjuvant chemotherapy. J. Clin. Oncol..

[27] Boellaard R., Delgado-Bolton R., Oyen W.J., Giammarile F., Tatsch K., Eschner W., Verzijlbergen F.J., Barrington S.F., Pike L.C., Weber W.A. (2015). FDG PET/CT: EANM procedure guidelines for tumour imaging: Version 2.0. Eur. J. Nucl. Med. Mol. Imaging.

[28] Haque W., Verma V., Hatch S., Suzanne Klimberg V., Brian Butler E., Teh B.S. (2018). Response rates and pathologic complete response by breast cancer molecular subtype following neoadjuvant chemotherapy. Breast Cancer Res. Treat..

[29] Viallon V., Latouche A. (2011). Discrimination measures for survival outcomes: Connection between the AUC and the predictiveness curve. Biom. J..

[30] Empereur-Mot C., Guillemain H., Latouche A., Zagury J.-F., Viallon V., Montes M. (2015). Predictiveness curves in virtual screening. J. Cheminform..

