# Supplementary material for: Clinical Utility of Pre-Therapeutic [18F]FDG PET/CT Imaging for Predicting Outcomes in Breast Cancer
Source: J Clin Med. 2023 Aug 24;12(17):5487. doi: 10.3390/jcm12175487 (PMC10488013; doi:10.3390/jcm12175487)
Supplement: Supplementary file 1 [file jcm-12-05487-s001.zip › jcm-2498192-supplementary.pdf]

Supplementary Materials

Supplemental table

Supplement table S1 : Distribution of PET imaging biomarkers 2
Supplement table S2 : Comparison of clinicopathological characteristics and PET imaging characteristics between patients with pathological complete response and no-pathological complete response 3
Supplement table S3 : Logistic regression analysis of biomarkers associated with no-pCR in cohort 1 5
Supplement table S4 : Logistic regression analysis of biomarkers associated with no-pCR in cohort 2 7
Supplement table S5 : Prognostic significance of biomarkers for 3-year RFS in univariate and multivariate analyses (Cox models) with thresholds value of TMTV equal to 28.1 cm^3 9
Supplement table S6 : Test for additional prognostic value of TMTV 10

Supplemental figure

Supplemental figure S1 : Distribution of the different molecular subtypes 2
Supplemental figure S2 : ROC curve to determine the best cut off value of TMTV to predict pCR in the whole cohort 4
Supplemental figure S3 : ROC curve to determine the best cut off value of TMTV to predict pCR in cohort 1 4
Supplemental figure S4 : ROC Curve to determine the best cut off value for TMTV to predict pCR in cohort 2 6
Supplemental figure S5 : Time dependent ROC curve for TMTV to predict 3 years RFS 8
Supplemental figure S6 : Predictiveness curve for TMTV for 3-years RFS 8
Supplemental figure S7 : Kaplan Meier curves according to molecular subtype 11

Supplemental figure S1 : Distribution of the different molecular subtypes

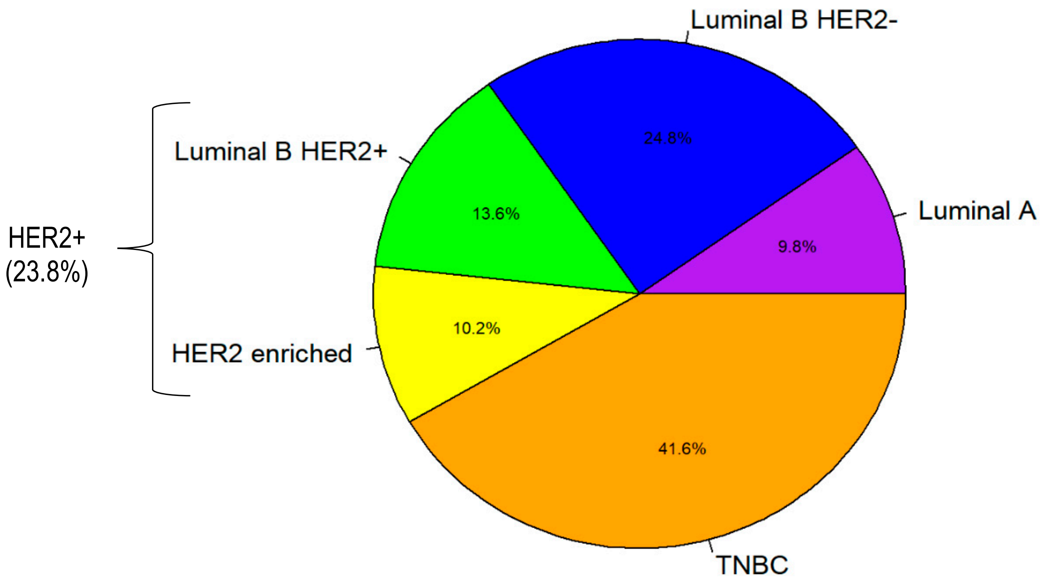

Supplement table S1 : Distribution of PET imaging biomarkers

	**Luminal**	**HER2 +**	**TNBC**
	Median [range]		
Tumor glucose uptake			
Tumor SUVmax	9.1 [6.2-13.1]	9.7 [6.3-12.9]	13.6 [9.0-17.3]
Metabolic tumor burden (cm^3)			
TMTV	10.9 [6.3-23.9]	9.6 [3.7-23.1]	9.2 [4.2-25.2]

Abbreviations: triple negative breast cancer (TNBC), maximum standard uptake value (SUVmax), tumor metabolic tumor volume (TMTV)

Supplement table S2 : Comparison of clinicopathological characteristics and PET imaging characteristics between patients with pathological complete response and no-pathological complete response

N=286		No-pCR<br>N= 174	pCR<br>N=112	
		**Mean (±SD), n (%)**		**p value**
Clinicopathological characteristics				
**Age**		**50.3 (±12.7)**	**47.2 (±11.7)**	**0.04**
**T stage**				**0.02**
	1	17 (9.8)	9 (8.0)	
	2	86 (49.4)	76 (67.9)	
	3	53 (30.5)	22 (19.6)	
	4	18 (10.3)	5 (4.5)	
**N+**		**114 (65.5)**	**52 (46.4)**	**< 0.01**
**Subtype**				**< 0.01**
	Luminal A	26 (14.9)	2 (1.8)	
	Luminal B HER2 -	52 (29.9)	19 (17.0)	
	HER2+	34 (19.5)	34 (30.4)	
	TNBC	62 (35.6)	57 (50.9)	
Histologic parameters				
**Ki67 ≥ 20%**		**136 (78.2)**	**103 (92)**	**< 0.01**
Vascular invasion		18 (10.3)	7 (6.2)	0.33
**Grade**				**< 0.01**
	I	5 (2.9)	0 (0)	
	II	66 (37.9)	24 (21.4)	
	III	136 (78.2)	103 (92)	
PET imaging characteristics				
Tumor glucose uptake				
Tumor SUVmax		11.48 (±6.37)	12.04 (±6.56)	0.48
Metabolic tumor burden (cm^3)				
**TMTV**		**30.7 (±52.2)**	**14.4 (±19.7)**	**< 0.01**

Abbreviations: pathological complete response (pCR), tumor (T), node involvement (N), triple negative breast cancer (TNBC), maximum standardized uptake value (SUVmax), total metabolic tumor volume (TMTV)

Supplemental figure S2 : ROC curve to determine the best cut off value of TMTV to predict pCR in the whole cohort

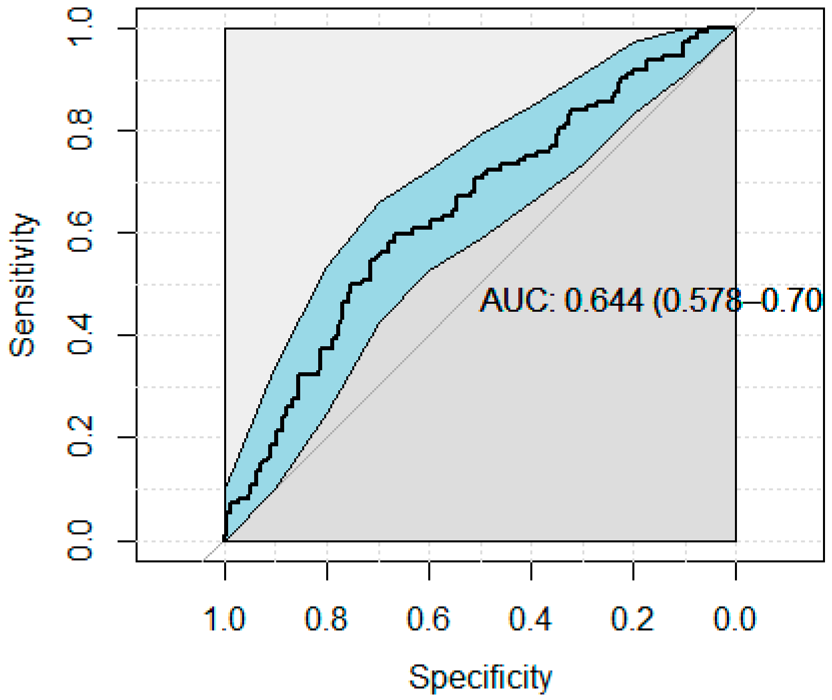

Supplemental figure S3 : ROC curve to determine the best cut off value of TMTV to predict pCR in cohort 1

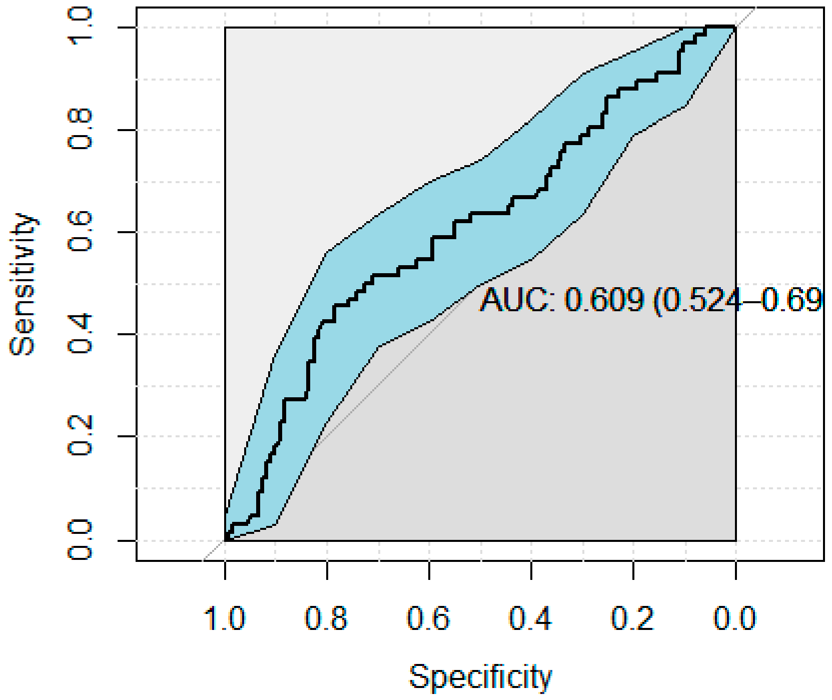

Supplement table S3 : Logistic regression analysis of biomarkers associated with no-pCR in cohort 1

Factor associated with no-pCR after NACT				
n=201	**Univariate**		**Multivariate**	
Events = 77	**OR (95% CI)**	**p value**	**OR (95% CI)**	**p value**
Age < 40 years (vs ≥ 40)	0.8 (0.4-1.5)	0.50	-	-
T stage 3-4 (vs 1-2)	1.9 (1.0-3.6)	0.05	1.8 (0.8-4.0)	0.13
N+ (vs N-)	2.1 (1.1-3.8)	0.02	-	-
**Subtype**			-	**< 0.01**
Luminal	1.0 (Reference)	-	-	
HER2+	0.2 (0.1-0.6)	< 0.01	0.2 (0.1-0.6)	-
TNBC	0.4 (0.2-0.8)	0.01	0.6 (0.3-1.3)	-
Vascular invasion (yes vs no)	1.5 (0.6-4.9)	0.40	-	-
Histologic grade 3 (vs 1-2)	2.5 (1.3-5.0)	< 0.01	-	-
**Ki67 ≥ 20% (vs < 20%)**	**0.3 (0.1-0.7)**	**0.02**	**0.2 (0.1-0.7)**	**< 0.01**
**TMTV > 6.3cm³ (vs ≤ 6.3cm³)**	**3.0 (1.6-5.8)**	**< 0.01**	**2.5 (1.2-5.3)**	**0.01**

Abbreviations: odd ratio (OR), confidence interval (CI), pathological complete response (pCR), neoadjuvant chemotherapy (NACT), tumor (T), node involvement (N)), triple negative breast cancer (TNBC), total metabolic tumor volume (TMTV)

Supplemental figure S4 : ROC Curve to determine the best cut off value for TMTV to predict pCR in cohort 2

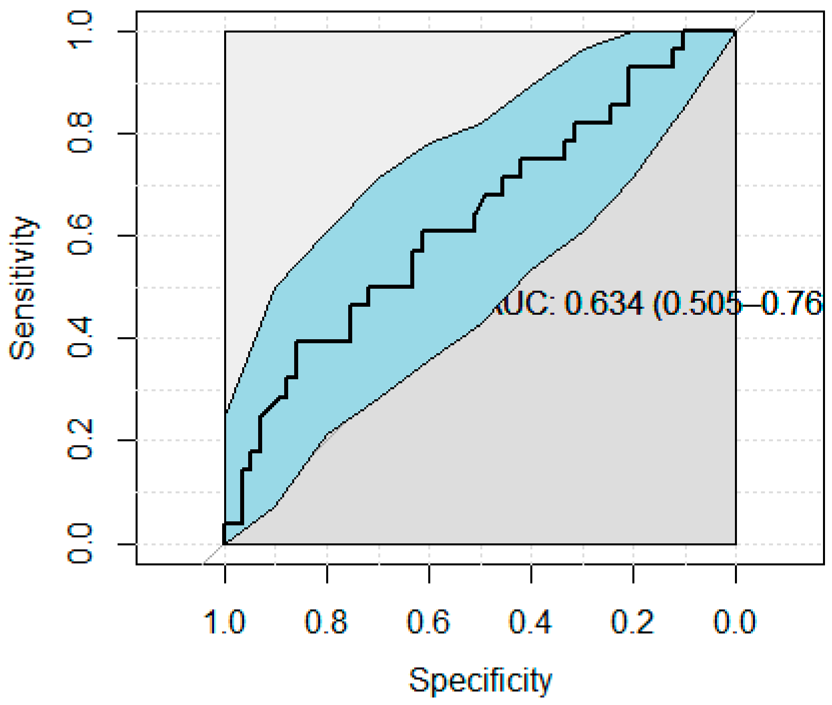

Supplement table S4 : Logistic regression analysis of biomarkers associated with no-pCR in cohort 2

**Factor associated with no-pCR after NACT**				
n=85	**Univariate**		**Multivariate**	
Events = 35	**OR (95% CI)**	**p value**	**OR (95% CI)**	**p value**
Age < 40 years (vs ≥ 40)	0.7 (0.2-1.9)	0.50	-	-
T stage 3-4 (vs 1-2)	1.4 (0.5-4.0)	0.50	-	-
N+ (vs N-)	2.1 (0.9-5.6)	0.11	-	-
**Subtype**			-	**< 0.01**
Luminal	1.0 (Reference)	-	-	
HER2+	0.2 (0.0-0.8)	0.05	0.2 (0.1-0.8)	-
TNBC	0.1 (0.0-0.3)	< 0.01	0.1 (0.0-0.4)	-
Histologic grade 3 (vs 1-2)	0.3 (0.1-1.0)	0.06	-	-
Ki67 ≥ 20% (vs < 20%)	0.2 (0.1-0.9)	0.07	-	-
**TMTV > 2.7cm³ (vs ≤ 2.7cm³)**	**3.9 (1.4-11.9)**	**0.01**	**3.6 (1.1-12.3)**	**0.03**

Abbreviations: odd ratio (OR), confidence interval (CI), pathological complete response (pCR), neoadjuvant chemotherapy (NACT), tumor (T), node involvement (N)), triple negative breast cancer (TNBC), total metabolic tumor volume (TMTV)

Supplemental figure S5 : Time dependent ROC curve for TMTV to predict 3 years RFS

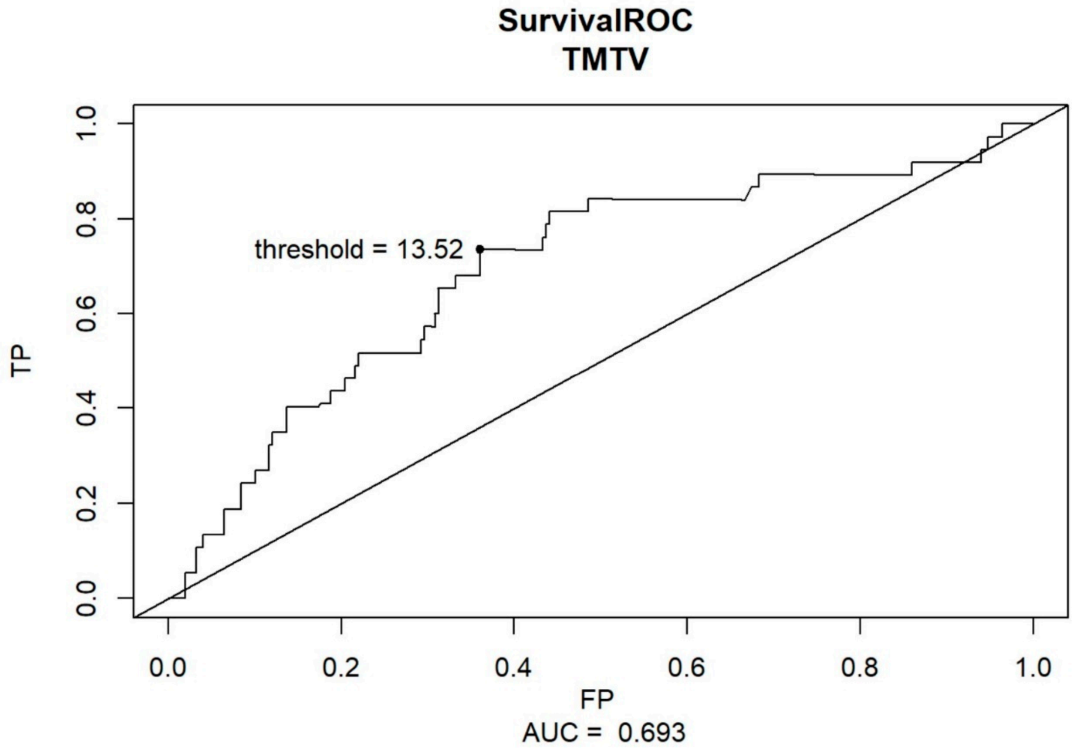

Supplemental figure S6 : Predictiveness curve for TMTV for 3-years RFS

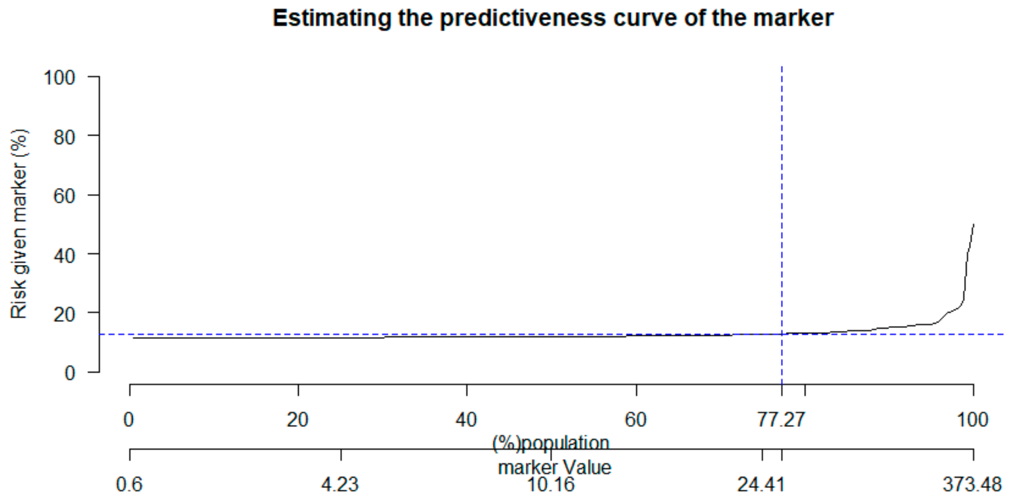

Supplement table S5 : Prognostic significance of biomarkers for 3-year RFS in univariate and multivariate analyses (Cox models) with thresholds value of TMTV equal to 28.1 cm³

3-YEAR RECURRENCE-FREE SURVIVAL				
n=286	**Univariate**		**Multivariate**	
Events = 65	**HR (95% CI)**	**p value**	**HR (95% CI)**	**p value**
Age < 40 years (vs ≥ 40)	1.1 (0.5-2.3)	0.78	-	-
pCR	0.5 (0.2-0.9)	0.04	0.5 (0.2-1.1)	0.08
T stage 3-4 (vs 1-2)	1.5 (0.8-2.9)	0.21	-	-
N+ (vs N-)	2.4 (1.1-5.1)	0.02	-	-
Molecular subtype				
Luminal	1.0 (Reference)	-	-	-
HER2+	0.3 (0.1-1.2)	0.09	-	-
TNBC	1.6 (0.8-3.2)	0.20	-	-
Vascular invasion (yes vs no)	1.7 (0.6-4.4)	0.26	-	-
Histologic grade 3 (vs 1-2)	0.8 (0.4-1.6)	0.55	-	-
Ki67 ≥ 20% (vs <20%)	2.4 (0.7-7.7)	0.15	2.4 (0.7-7.9)	0.15
**TMTV > 28.1 cm^3 (vs ≤ 28.1 cm^3)**	**3.1 (1.6-5.9)**	**< 0.01**	**2.6 (1.3-5.0)**	**< 0.01**

Abbreviations: hazard ratio (HR), confidence interval (CI), pathological complete response (pCR), tumor (T), node involvement (N), triple negative breast cancer (TNBC), total metabolic tumor volume (TMTV)

Supplement table S6 : Test for additional prognostic value of TMTV

	3-year RFS			
	TMTV > 13.5 cm^3 (vs ≤ 13.5 cm^3)		TMTV > 28.1 cm^3 (vs ≤ 28.1 cm^3)	
Cox model variables	LR	LR p value	LR	LR p value
Multivariable model <u>with</u> TMTV	23.9	-	15.5	-
Multivariable model <u>without</u> TMTV	12.9	< 0.01	12.9	0.11

Abbreviations: likelihood ratio (LR), recurrence free survival (RFS), total metabolic tumor volume (TMTV)

Explanation: The likelihood ratio test (LRT) for the added prognostic value of imaging biomarkers was obtained by comparing the log-likelihoods of the multivariable Cox prognostic models with and without TMTV (chi-square test). The multivariable model included TMTV (> 13.5 cm3 vs ≤ 13.5 cm3) or TMTV (> 28.1 cm^3 vs ≤ 28.1 cm^3), Ki67 (≥20% vs <20%) and pathological complete response.

Supplemental figure S7 : Kaplan Meier curves according to molecular subtype

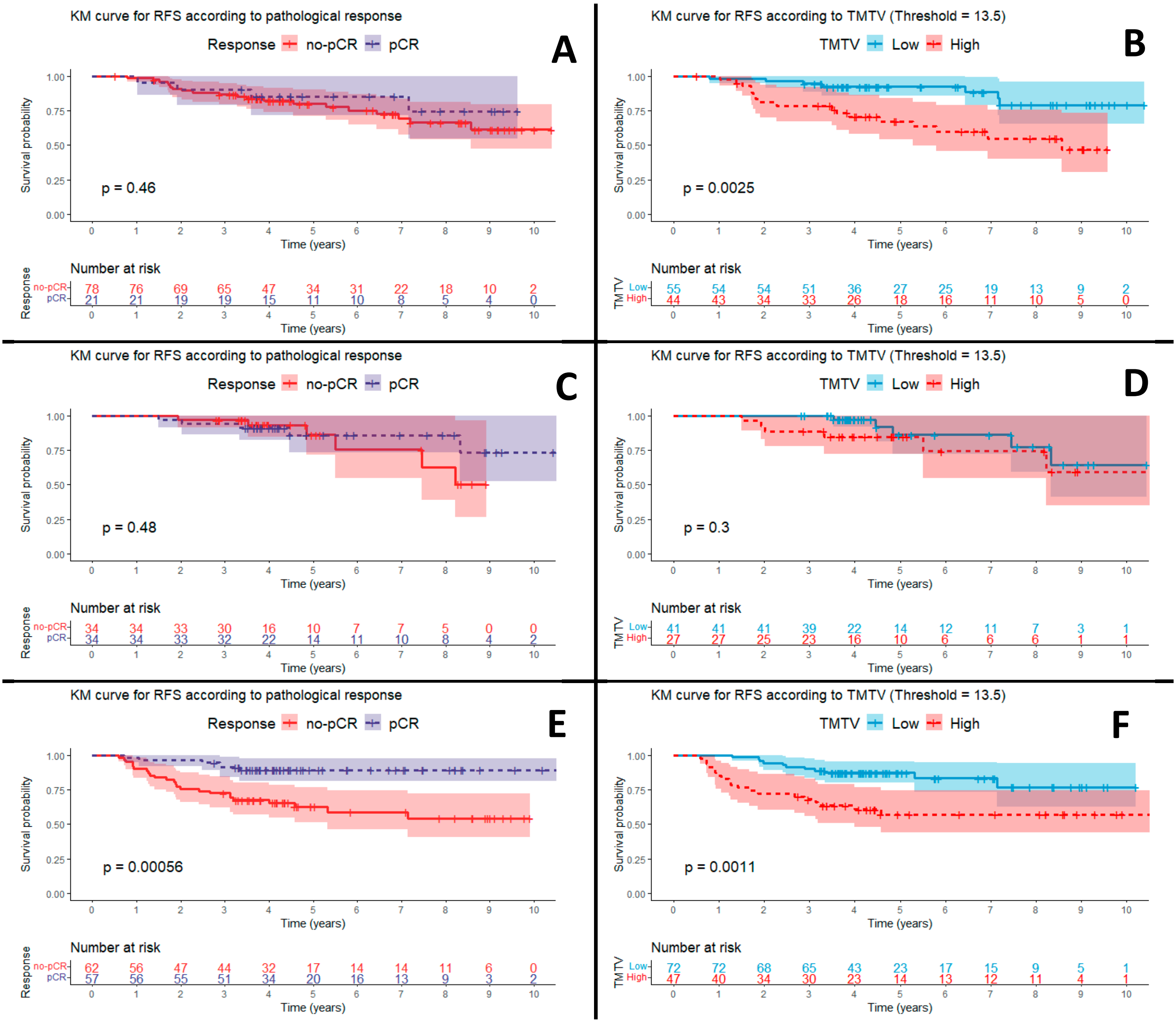

Legend: (A) Kaplan Meier curve according to pathological response for Luminal subtype, (B) Kaplan Meier curve according to TMTV for Luminal subtype, (C) Kaplan Meier curve according to pathological response for HER2 subtype, (D) Kaplan Meier curve according to TMTV for HER2 subtype, (E) Kaplan Meier curve according to pathological response for TNBC subtype, (F) Kaplan Meier curve according to TMTV for TNBC. The p values obtained are those of the log rank test.
